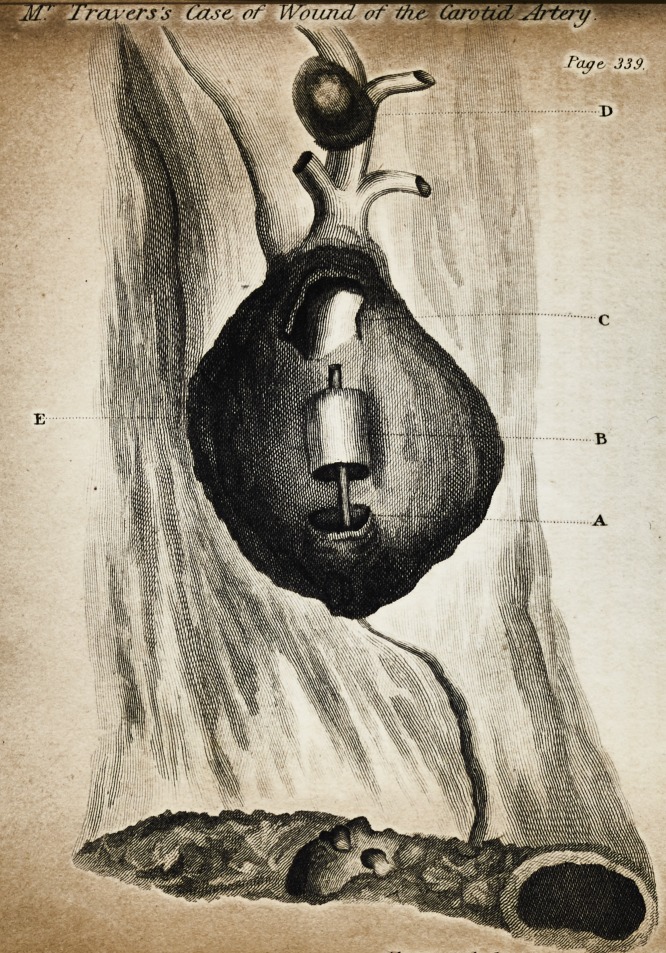# Case of Wound of the External Carotid Artery, behind the Angle of the Jaw, for Which the Common Carotid Artery Was Tied

**Published:** 1826-10

**Authors:** 

**Affiliations:** St. Thomas's Hospital


Mr. Travers's Case of Wound of the Carotid Artery.

### WOUND OF THE CAROTID ARTERY.

*Case of Wound of the external Carotid Artery, behind the angle of the Jaw, for which the common Carotid Artery was tied.* Treated at St. Thomas's Hospital, by Mr. Travers. *With Remarks.*

[with an engraving.]

On June 27th, 1826, —— Black, a muscular man, of respectable appearance, æt. about thirty-five, was brought to St. Thomas's Hospital, at seven a.m., with a small penetrating wound near the angle of the jaw, on the right side. It appeared that, in consequence of his creating a disturbance the preceding night at Vauxhall, while in a state of intoxication, he was conveyed to the watch-house, where he conducted himself in so outrageous a manner as to render it necessary to confine him in the cell. A short time afterwards, a watchman, looking in by accident, saw the floor covered with blood, which was flowing rapidly from a wound in the neck, supposed to have been inflicted with a penknife. The hemorrhage was checked by pressure with the finger, till the assistance of a surgeon was procured, when a piece of sponge was introduced into the wound, a bandage applied, and the man was conveyed to the hospital. No blood escaped during the interval, but, when admitted, he was in a state verging on syncope; the face and lips pallid; the extremities cold; pulse scarcely perceptible; and he was evidently delirious.

On removing the bandage and sponge, the hemorrhage became terrific, but was restrained by firm pressure on the wound. The wound, three-quarters of an inch in length, on the right side of the neck, was found to extend from the front edge of the sterno-mastoid muscle, rather below the lobule of the ear, obliquely downwards and forwards to the angle of the jaw. The finger, introduced into the wound, readily passed as far as the second joint in the direction of the base of the skull.

Pressure was continued until Mr. Travers arrived, who dilated the wound, and, having removed a large coagulum, endeavoured to discover the bleeding orifice, but without success, on account of the depth of the wound, and the obscurity of the parts from the continual accumulation of blood. Finding that the hemorrhage was restrained by steady pressure on the common carotid artery,

Mr. Travers resolved on securing that vessel. For this purpose, while Mr. SOUTH commanded the flow of blood, by introducing his finger into the original wound, and compressing the artery against the angle of the jaw on its inner side, Mr. Travers made an oblique incision from the lower angle of the wound to within one inch of the clavicle, along the anterior edge of the sterno-mastoid muscle. Turning aside the omo-hyoideus, and proceeding to open the common sheath of the vessels, the internal jugular vein was drawn aside, and a single ligature passed around the artery from the inner to the outer side, by means of the aneurism-hook, carefully excluding the par vagum. Immediately after tightening the ligature, a very feeble pulsation was perceived above it, and also a slight oozing of arterial blood; which ceased, however, after a few seconds. The edges of the integument were approximated by three stitches and adhesive plaster, and the whole covered with simple dressing. The patient was then carried to bed; when it was found necessary to confine his hands and feet, as he was highly delirious. He was ordered to have a plentiful supply of toast and water, and nothing more.

Four P.M.—Slight oozing of blood, but not in greater quantity than from a common wound of similar extent. Pulse 110, quick, small, and hard; extremities warm; some pain in the head; copious mucous expectoration. Is quiet, and perfectly sensible; but answers inquiries in a morose manner, and refuses to give any information as to his name or residence.*

28th, twelve M.—Slept three or four hours in the night. Countenance tranquil, and manner composed; pulse 100, hard, and sharp; skin natural; bowels have not yet been moved.

Capiat Ol. Ricini ℥ ss. statim.—The bandages to be removed from his arms and legs.

29th.—Two copious evacuations yesterday evening. A quiet night, and the bowels have acted again this morning. There is nothing remarkable in his general appearance.

30th.—In the afternoon of yesterday, a sudden alteration took place: he became excited in the highest degree, and, watching his opportunity, rushed past the attendants, and, ascending the gallery of the operating theatre, jumped from thence into the area, alighting on his feet; he then descended some steps of the great staircase, in his way striking at a man who attempted to stop him; and, placing his hands upon the balusters, leaped from a height of twenty feet and a half, and alighted on the stone pavement, with no other injury than a slight bruise of the back and graze of the right elbow. He was taken up and replaced in bed, when his hands and feet were again confined. He afterwards complained of thirst, and of severe pain in his back and loins, and in the head. He passed the night quietly, though without sleep. This morning

* It was shortly afterwards ascertained that he held the situation of chorus-master at Drury-lane Theatre, and was considered a person of some talent in his profession, but of a reserved and haughty disposition.

he again became violent, and, having succeeded in extracting one arm from the jacket, tore off the dressings from the wound; but, fortunately, without doing mischief. The wound has a very healthy appearance, secreting pus in considerable quantity. No adhesion has taken place, probably in consequence of his restlessness, as he continually moves his head in every direction. Bowels have not been moved to-day. Thirst continues.

Evening.—Head cool, slightly painful; skin of the natural heat, and perspirable; pulse seventy, soft, and full.

An injection administered, by which the bowels were moved once.

July 2d.—He was tranquil yesterday, without headache; skin cool; pulse seventy-two. He has passed a tolerably quiet night, but without sleep. This morning he is again delirious. Pulse 100, strong, and hard; tongue white; bowels confined. In the absence of Mr. Travers, he was visited by Mr. Green, who ordered eight ounces of blood to be drawn from his temples by cupping, ten grains of calomel to be taken immediately, and a cold lotion to his head. He appeared to be much relieved by the cupping. It is, perhaps, worthy of notice, that much greater difficulty was found in obtaining blood from the right side of the head, than from the left. The bowels were acted upon once.

In the evening, he was quite composed; ate some bread and milk; and afterwards slept for several hours.

3d.—Is in a state of extreme languor, answering questions with difficulty, and much exertion. The countenance is sunk; lips and complexion pallid; disposition to coma; pulse seventy-two, full, and soft. A dessert-spoonful of port-wine was given, diluted with water: this had the effect of increasing the sharpness of the pulse, but produced no other alteration in the symptoms. The wound is healthy, granulating, and discharging copiously.

Inf. Cascar. ℥ x.; Acid. Sulph. dil. m. xij.; Syr. Aurant. ʒ j. M. sextis horis sumend.—Arrow-root.—Vini rubri ℥ ij. quotidie.

5th.—There is a decided improvement since the last report, both in the general symptoms and in the countenance. Wound granulating, discharge copious; bowels confined.

Ol. Ricini ℥ss. statim et p. r. n. repetend.; adde sing. Haust. Tr. Cinch. ʒj.

9th.—The ligature came away this morning. Though generally quiet, there is again a wildness about his countenance and manner, which indicates the necessity of continuing the confinement of his limbs. The wound is filling by granulation; the discharge copious, but healthy. Has some appetite, and takes broth, bread and milk, &c. with relish.

12th.—Again had a paroxysm of delirium, in which he tore off the dressings, producing a slight discharge of blood from the wound. His appetite is good, and he is free from pain. Refuses to take the tonic, which is therefore discontinued. Bowels still confined.

Small doses of the Ol. Ricini every other day. Meat once daily.

15th.—Had a cold shivering fit yesterday afternoon, which lasted half an hour, and which he attributed to a current of air from the window by his bedside, which had been open rather later than usual the evening before. The granulations are pale and languid; the discharge copious.

Dec. Cinchon. ℥x.; Acid. Sulph. dil. m. x.; Tr. Cinchon. ℥j. M. ter die sumend.

19th.—Has continued to improve progressively since last report.

A solution of Sulphate of Copper to be applied to the wound daily before dressing.

27th.—Tongue clean; pulse ninety, small, and regular; appetite good; bowels open; takes Mist. Sennæ Comp. p.r.n.; sleeps well; head quite free from pain; complains of soreness in the feet and legs when he walks, and when the legs are hanging down. Wound healing; discharge still copious.

Frictions with Linim. Saponis comp. c. Opio, and rollers to the feet and legs.

August 8th.—Since last report he has been daily walking about the ward. Yesterday afternoon he had another slight shivering fit. He now complains of pain in the head, and in the right side of the neck, which is swollen and tender. The incision is healed, except about a quarter of an inch at the lower part of the wound, from which the discharge continues copious. Tongue white; appetite bad; considerable thirst; bowels tolerably regular.

9th.—℞. Mist. Ammon. Acet. c. Liq. Antim. Tart. ter die sum.—Mist. Sennæ Comp. alt. aur.—Catap. Lini.

12th.—The opening in the wound is now deep and nearly circular; discharging copiously. The swelling and pain have abated; appetite returning.

18th.—At twelve last night, hemorrhage, to the amount of about eight ounces, took place in the poultice: the patient, by making pressure with his own finger easily prevented the further loss of blood. The dresser was called, who applied a compress and bandage.

At half-past four A.M. a second hemorrhage came on, and about the same quantity was lost. Compress again applied, with the effect of arresting the bleeding. For the last two or three days, he has had pain in the right side of the neck and face, and his rest has been disturbed, which he attributed to the irritation of a decayed stump in the upper jaw, which he himself extracted. Fancies that he has taken cold, and desires to have additional clothing.

19th.—Pulse eighty-eight, feeble, but sharp; no pain; skin cool and moist; furred tongue; bowels open; sleeps indifferently well. Countenance sunk and pallid, with a marked expression of anxiety.

20th.—Bleeding recurred four times in the course of the day, but, the hemorrhage being easily checked by pressure with the finger, very little blood was lost.

21st.—Wound bled three times. Head rather hot.

Apply cold lotion.

22d.—It is now found necessary to keep up constant pressure with the finger, as the hemorrhage recurs on the instant of its removal. One or two ounces of blood may have been lost in the course of the day. He lies on his back, with the shoulders considerably elevated, as a more recumbent posture induces a painful affection of the head. A very peculiar dazzling brightness of the eye is observable.

23d.—Has lost, perhaps, three or four ounces of blood since yesterday, which has escaped when the thumb has been removed, as in changing assistants. Pulse 100, small, but not more feeble than for several days past. The wound is now considerably enlarged.

Two P.M.—It was resolved, in consultation, to endeavour to find the bleeding orifice, and to tie it. In pursuance of this resolution, he was conveyed to the theatre. On removing the coagulum, which was already half extruded, at least two pounds of florid blood escaped, in a full, uninterrupted stream, in less than two minutes. By thrusting a sponge into the upper part of the wound, and making firm pressure upon it, the hemorrhage was repressed. The countenance instantly assumed a ghastly hue; respiration became difficult and laborious; the eyes were elevated, and symptoms of approaching dissolution made their appearance. Wine and brandy were repeatedly administered, without effect, and at half-past three P.M. he expired.

*Sectio cadaveris, nineteen hours after death.*—On extending the wound in the direction of the cicatrised incision, the cavity of an abscess was found, to which the coagulum removed yesterday accurately corresponded. It was about the size of a pullet's egg. At the bottom of this cavity was seen a portion of the entire cylinder of the carotid artery, about one-third of an inch in length, insulated, both above and below, by an intervening space of about one-third of an inch. The orifice of the lower portion of the carotid was circular, but its calibre diminished; and, on slitting it open, it was found that a plug of adhesive matter filled the vessel, and perfectly secured it from hemorrhage. Nearer the heart was a long and firm coagulum of blood. The orifice of the upper part of the trunk was irregular from ulceration, having a tongue-like prolongation, formed of the posterior third of its parietes. This upper portion of the vessel, to which the coagulum was exactly moulded, was patulous; and from this the blood had flowed in the fatal hemorrhage, by reflux through the opposite carotid and vertebrals. On examining the artery at the site of the original wound, an aneurismal pouch, as large as a sparrow's egg, was found opposite the origin of the facial artery. In the viscera, the only circumstance worthy of notice was an enlargement of the pineal gland, to about double its ordinary size.

REMARKS.—This case is valuable, as it establishes the important fact that a ligature of the common carotid trunk is available to arrest hemorrhage from a wound of the external carotid artery, so situated as not to admit of being included between two ligatures. The secondary hémorrhage, which proved fatal *at the expiration of ten weeks* from the injury, does not in the remotest degree impugn the accuracy of this conclusion. The ulceration of the entire circle of the artery, in common with the surrounding parts, which induced the fatal hemorrhage, was plainly owing to the formation of an extensive abscess; a contingency which probably would not in another instance occur, or the occasion of which (more correctly speaking) might be guarded against: viz. the disturbance of parts inseparable from a long and fruitless search after the wound of the artery. Fruitless, even should the spot be ascertained, if its situation precludes the application of a ligature above the wound; and, if it should not be so situated, it amounts to a question whether the employment of a second ligature offers a paramount advantage to the risk incurred in the elaborate dissection necessary to detect and expose the wound of a deep artery, in such most unfavourable circumstances.

In the case above detailed, had the operation of tying the carotid trunk been practised distinct and at a distance from the original wound, and had this remained undisturbed, it is extremely probable that the coagulum would have been gradually absorbed; that suppuration of the cavity would never have taken place, or have been of very inconsiderable extent; and that the case would have terminated, like that of aneurism, with the separation of the ligature. This is a reflection which, candid persons will be ready to admit, was neither likely to have suggested itself to this extent in the first instance, nor perhaps, if it had, would it have formed, without a precedent, a vindicable ground of procedure. Such after-reflections, however, suggested by experience, constitute the basis of scientific improvement, and therefore ought to be candidly received. If there is reason to believe that the situation of the wound in an artery is such as to preclude the possibility of including it between two ligatures,—and if there is also reason to believe that the successful application of one ligature depends upon the non-disturbance of parts, then it is clear that the wound should be treated as the aneurismal sac, at a distance, by simply taking off the direct impulse of the current of blood, leaving the wound, as the aneurism, undisturbed.

Another view of this question appears to lend confirmation to the practice proposed. The puncture of an artery is difficult to be discovered,—much more difficult than its division, notwithstanding the retraction in the latter case; unless, indeed, time sufficient has elapsed since the division to have given the wound a coating of adhesive matter, when the detection of the arterial mouth is often extremely embarrassing.

In the case of a truncated artery on the surface of a stump, the stream of blood is a direction to the mouth of the vessel; but the mode in which blood escapes from a lateral wound or puncture of an artery, is such as to give a very indirect, or even erroneous clue, to its precise situation. A somewhat extended and clean exposure of the vessel by dissection is necessary to demonstrate a puncture or incision, when we are aided by the quietude of the parts in death, and no longer incommoded by the gush of blood. But the circumstances attending a penetrating wound tend as much as possible to obscure it. The resistance opposed by the sheath, the cellular membrane, the muscles, and fascia, is exhibited in the formation of a cavity, loaded with coagula, between the wound of the artery and that of the integument, from which the artery is entirely shut off, except at the point from which the blood enters it; and even here the communication is oblique or valvular, owing in part to the direction and force of the current, but especially to the infiltration of the sheath and connecting membrane, and the altered relative position of the two (outer and inner) wounds. Hence the difference observed in the mode of escape of blood from the wound of an exposed artery, and of one which is covered and concealed from view. In the former, the blood, meeting no obstacle, continues to be delivered, as at first, in a distinct saltatory jet: in the latter, though it flows *per saltem* at the instant of the wound, the jet almost immediately ceases, and, if the hemorrhage continues or recurs, the blood accumulating in the wound is discharged at intervals; proving at the same time the existence and the partial efficiency of the natural checks above described ; and this happens, however freely the external wound is dilated. When this is done, and the coagula cleared away, the bleeding seems to arise, not from a point, but from the whole bed of the wound. It is, in fact, the overflowing of a chamber or reservoir, which is supplied, as it has been formed, by the interrupted delivery of the blood from the hidden orifice of the vessel.

In wounds not admitting of ocular demonstration, and the application of including ligatures, as in the case above detailed, a ligature on the side next the heart might, *a priori*, be supposed an insufficient security against hemorrhage by reflux of the circulation; in the arteries of the head and extremities especially. It was, however, found sufficient to command the hemorrhage, in this and another case of wounded carotid; and it has proved sufficient in punctured wounds of the brachial and femoral arteries. The Baron

Dupuytren recently mentioned to the writer of these remarks a case, in which the popliteal was successfully tied for a wound of the posterior tibial artery. Wounds of the arterial trunks of the arms and legs are generally supposed to require a ligature to be placed on either side of them. This, as regards primary hemorrhage, must depend on the size of the wound. If a puncture of the carotid, large enough to occasion a hemorrhage which had nearly proved fatal, (and which, left to itself, undoubtedly would have been fatal in a very few hours,) requires only one ligature for its complete restraint, *a fortiori* a puncture of either artery of the arm or leg might be safely so treated. The division of the carotid is fatal on the spot, so that no parallel case can be put; but a semi-division, or a complete division, of the posterior tibial, or ulnar artery, cannot be left to a single ligature with safety. But there is a sufficiently cogent reason why a second ligature should be employed, even though the primary bleeding be commanded by one, where a wounded artery is superficially seated, and consequently the danger of inducing abscess and secondary hemorrhage, by separation of deep-seated parts, does not exist. This is the prevention of what may be called traumatic aneurism; for it is shown in the foregoing case how soon, after it had failed to maintain the hemorrhage, a sufficient impulse was given by the refluent column of blood to convert the newly-cicatrised wound into an aneurism; though it is doubtful if an aneurism so formed would increase or become formidable, it were surely wise, when practicable, to anticipate its production.

In all cases, the danger of secondary hemorrhage from ulceration is in proportion to the disturbance of parts: when, therefore, primary hemorrhage from a deep-seated artery is commanded by compression of the trunk nearer to the heart, it will be a safer and a better practice to apply a ligature distinctly, as in aneurism, leaving the wound to itself. When, on the contrary, the vessel is superficially seated, it will be better to secure the wound between two ligatures, even though it should not be necessary to do so for the command of the primary hemorrhage.

The writer remembers the failure of an attempt to cure an aneurism of the anterior tibial artery, by a ligature above the sac: the pulsation, at first arrested, or more probably unperceived, returned vigorously in a day or two, and the operator was compelled to apply a second ligature below the sac. This is not at variance with the fact mentioned by M. Dupuytren, because the reduced force of the circulation would allow of the adhesive process in the case of a recent wound, provided the parts were not disturbed, so as to provoke suppuration.

The proximity of the wound or aneurism of the carotid, and of the arteries of the arms and legs, to a natural boundary of the circulation, and the peculiar mode of communication, by cross branches, subsisting between the carotids and vertebrals, and the ulnar and radial and two tibial arteries, makes the employment of a second ligature an analogous, if not parallel, case—circumstances as regards the extent of the wound, &c. being, as far as the case admits, the same. And the same general principle seems applicable to the treatment of wounds and aneurisms, both of which are formidable in proportion to the vigour of the circulation through the sac or wounded vessel respectively. It is not necessary to the cure of aneurism, nor the arrest of hemorrhage, that the blood should be excluded, if the impetus of the circulation be so taken off the sac or wound, that the pulsation in the one case, and the hemorrhage in the other, cease, from the rapid conversion of the fluid into a solid. But it would be extraordinary if the digital portion of an artery of the limbs, as the ulnar or tibial, required a ligature for either wound or aneurism, when it is found unnecessary to secure the cranial portion of the carotid, for a wound or aneurism of that artery. That the regurgitation should be sufficiently profuse and powerful in the completely open state of the carotid artery, when divided by ulceration, to extinguish life at once, cannot be surprising, when we consider the diameter of that vessel, its origin, and the direct channels of communication between the principal branches of the internal carotids and vertebrals (anterior et posterior cerebri,) forming the circulus arteriosus at the basis of the brain. The most abundant anastomotic communication elsewhere is not to be compared with this.

The compound formation of the palmar and plantar arches by direct communication, is, in like manner, a sufficient explanation of the recurrent force of the circulation, and the necessity for a ligature on the lower portion of the vessel. If the communication were exclusively and distinctly anastomotic, it would probably be so much feebler as to render this unnecessary.

An instrument, resembling in its outline a button-hook, used for conveying the ligature around the artery, on this and several other occasions at St. Thomas's Hospital, having an oblong eye at its extremity, which is flattened and pointed, will be found greatly more convenient than the common aneurism needle. Its short curve fits it to embrace the cylinder of the vessel, as its thin point separates the sheath in its passage. It was designed and first used by Mr. Green.

*Explanation of the Plate.*—A, the lower orifice of the carotid artery; B, the detached portion; C, the upper orifice; D, the aneurismal bag on the site of the wound; E, the cavity from which the coagulum was removed.

## Figures and Tables

**Figure f1:**